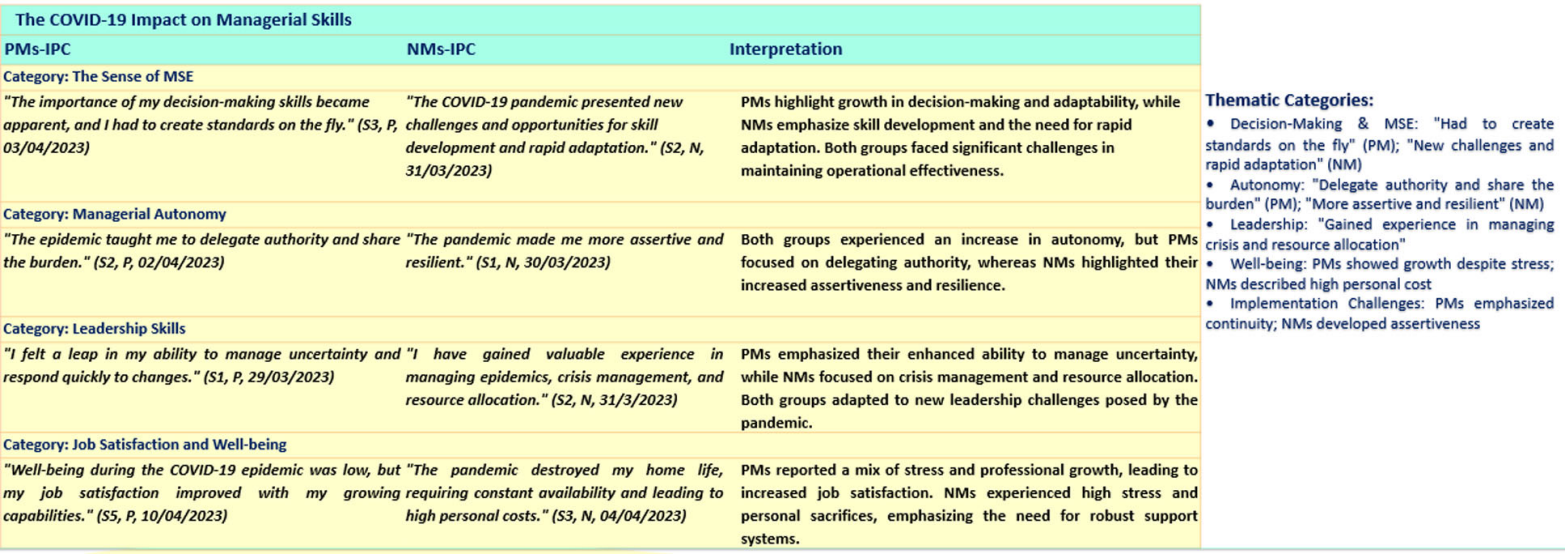# 171 Rapid Patient Isolation Precautions Reconciliation via Technology Enabling Tool Comparing Nursing Documentation with Surveillance Data

**DOI:** 10.1017/ash.2026.10572

**Published:** 2026-06-23

**Authors:** Dafna Chen, Stefan Cojocaru

**Affiliations:** 1 National Center for Infection Prevention and Antimicrobial Resistance, Israeli Ministry of Health; 2 Alexandru Ioan Cuza University from Iasi, Romania

## Abstract

**background:** the covid-19 pandemic placed infection prevention and control (ipc) units at the center of hospital crisis response. beyond operational overload, ipc managers were required to make rapid decisions, adapt standards in real time, and lead teams under sustained uncertainty. these conditions highlighted the importance of managerial autonomy, self-efficacy, and leadership, and provided a unique opportunity to examine how extreme crisis reshapes managerial capacity in practice. objectives: to examine how the covid-19 pandemic influenced managerial self-efficacy, autonomy, and leadership among ipc physician managers and ipc nurse managers, and to explore associations between these managerial traits and ipc implementation-related activities during the pandemic. **Methods:** a mixed-methods study was conducted across israeli public hospitals. the quantitative component included 50 senior ipc unit managers(19 physician managers and 31 nurse managers) from 29 of 31 israeli public hospitals, reflecting broad national representation(table 1). the qualitative component included semi-structured interviews with 10 ipc managers(5 physicians and 5 nurses) from 9 hospitals. study instruments included the managerial self-efficacy questionnaire (α=0.91–0.97), managerial autonomy questionnaire (α=0.68–0.89), leadership evaluation questionnaire (α=0.65–0.97), organizational change implementation questionnaire (α=0.78–0.92), and the covid-19 managerial impact questionnaire (α=0.86–0.94). all instruments underwent pilot testing and expert review to ensure content validity and reliability. quantitative data were analyzed using analysis of variance and pearson correlation coefficients, while qualitative data were analyzed thematically to capture experiential and contextual insights. **Result:** mean managerial scores during the pandemic were high and comparable between physician and nurse managers across autonomy, conflict management, leadership during crisis, and overall managerial performance, with no statistically significant differences between groups (figure 1). perceived covid-19 impact demonstrated significant positive correlations with multiple managerial traits, including autonomy (r=0.473), self-efficacy (r=0.388), strategy and vision (r=0.458), process management and intervention boundaries (r=0.463), and transformative leadership (r=0.320) (p<0.05)(figure 3). correlations were also observed between covid-19 impact and core ipc activity domains, particularly research, consultation, training, and infection monitoring, while weaker or negative associations were noted for investigative and event-driven activities(figure 2). qualitative findings reinforced these patterns, revealing accelerated decision-making capacity, increased managerial autonomy, and strengthened leadership identity. physician managers emphasized maintaining operational continuity, whereas nurse managers highlighted increased assertiveness alongside substantial personal and emotional burden. **Conclusion:** the covid-19 pandemic functioned as a catalyst that reshaped managerial skills in ipc units, strengthening autonomy, self-efficacy, and leadership under crisis conditions. while managerial growth was evident, the findings also underscore the personal cost borne by ipc leaders, particularly nurses. investment in leadership development and organizational support systems

**Figure:**
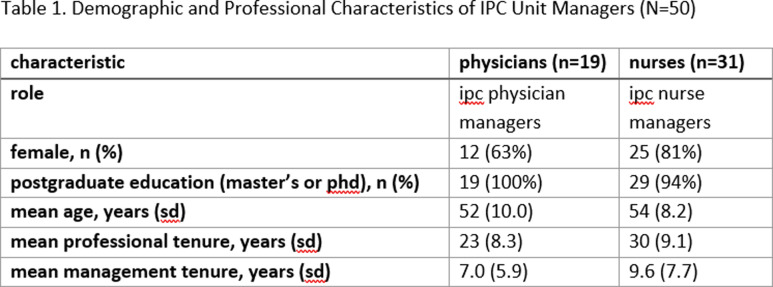

**Figure:**
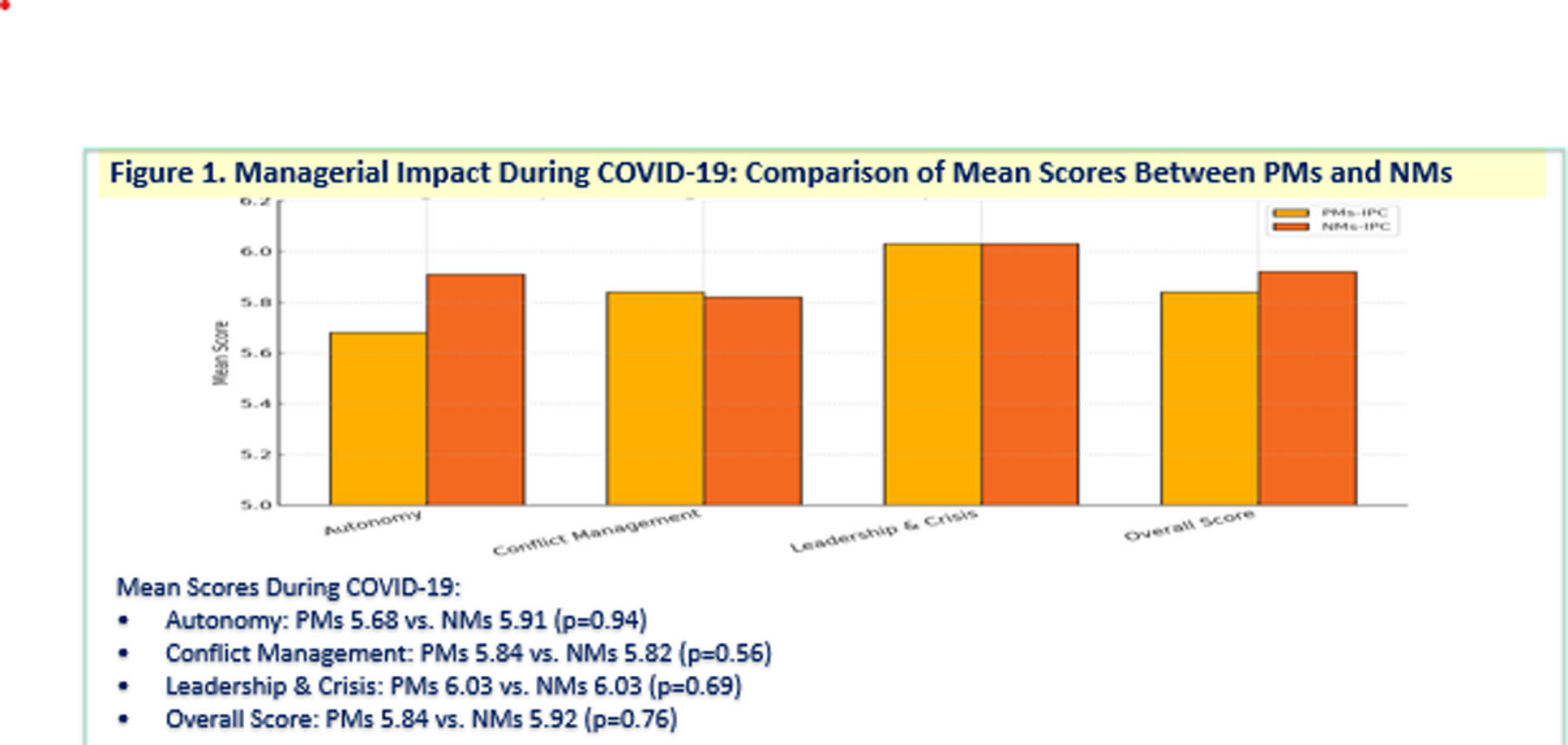

**Figure:**
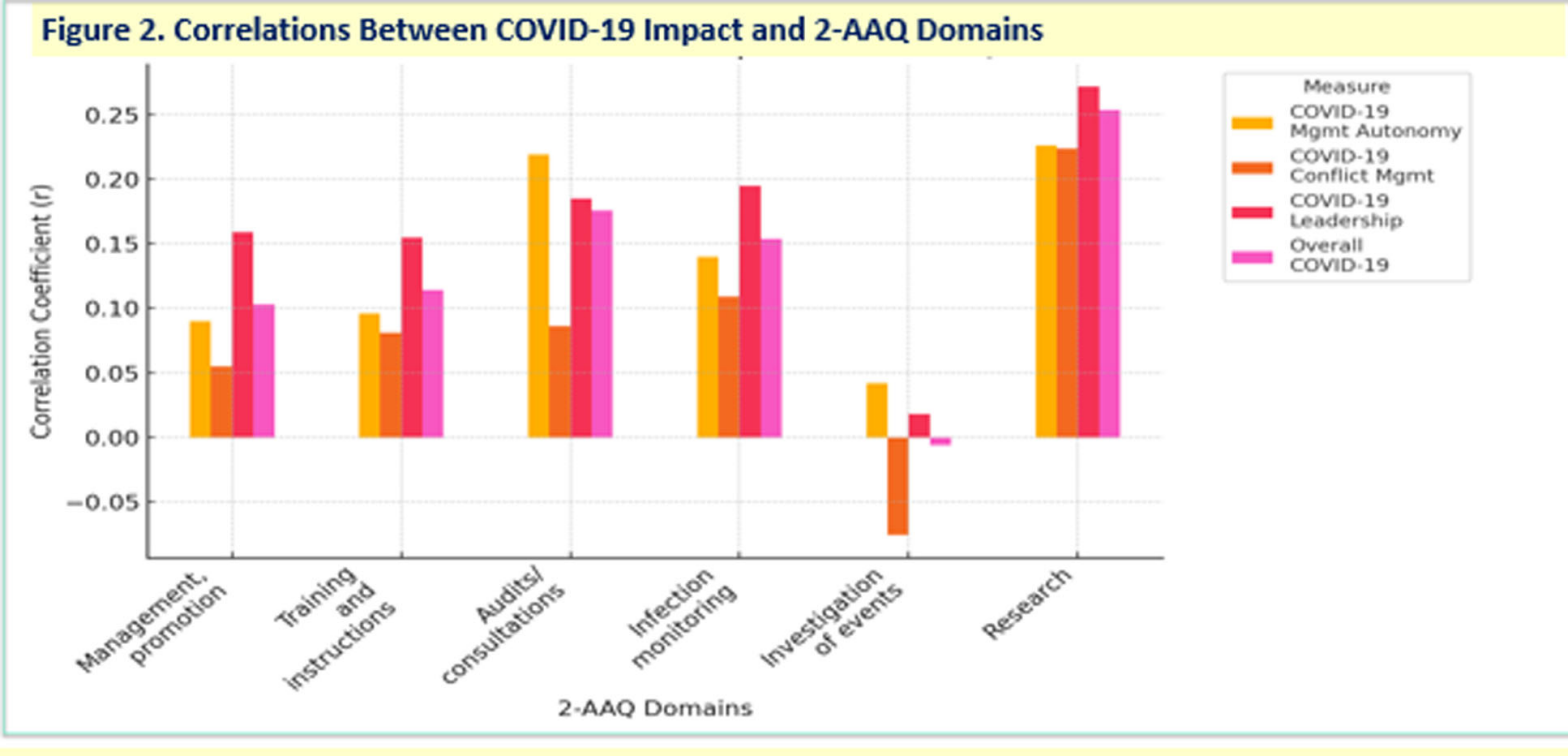

**Figure:**
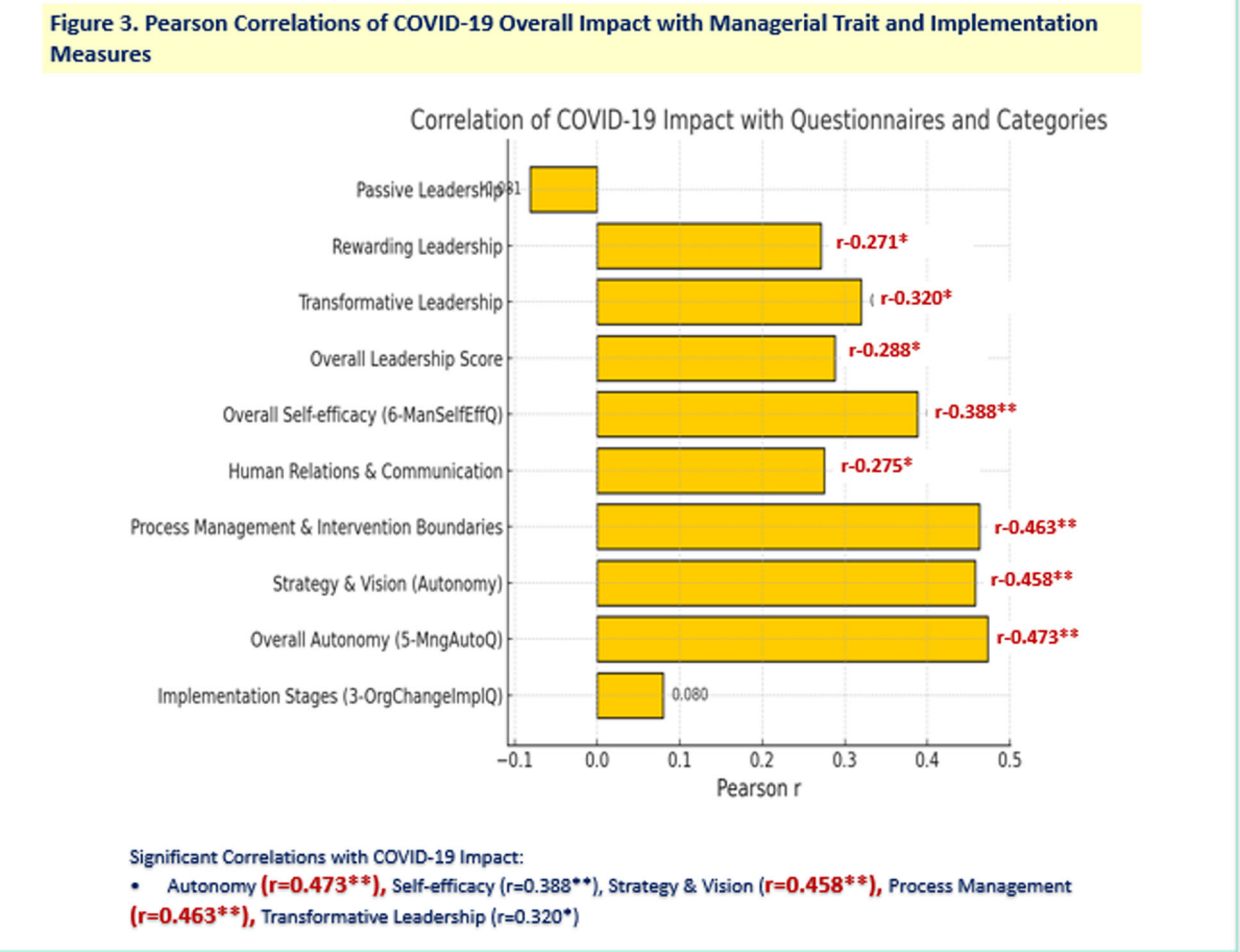

**Figure:**